# “What Society Expects of Healthcare and the Picture That We Paint”—A Qualitative Piece in the Puzzle to Inform Implementation: What Do People Using Services and Healthcare Professionals Look for in Questionnaires to Support Person-Centred Care in Physiotherapy and Podiatry Outpatients?

**DOI:** 10.3390/healthcare14213778

**Published:** 2026-11-05

**Authors:** Katherine E. Woolley, Robert Letchford, Kathleen Withers

**Affiliations:** 1 CEDAR (Centre for Healthcare Evaluation, Device Assessment and Research), Cardiff and Vale University Health Board, Cardiff CF14 4UJ, UK; katherine.woolley2@wales.nhs.uk; 2 Welsh Value in Health Centre, Value Transformation, NHS Performance and Improvement, Cardiff CF10 4BZ, UK; robert.letchford2@wales.nhs.uk; 3 School of Medicine, Cardiff University, Cardiff CF14 4YS, UK

**Keywords:** person-centred care, patient activation, patient agency, health literacy, physiotherapy, podiatry

## Abstract

Background/Objectives: Directed questionnaires (e.g., patient-reported outcomes, measuring patient activation) can support equal partnership in a person-centred model of care. Considering stakeholder preferences is essential for effective implementation to prevent high burden and low engagement from individuals using healthcare services. We examined (i) the perception of tools that can be used to measure empowerment and self-efficacy and support person-centred care among healthcare professionals (physiotherapists and podiatrists) and people using services within physiotherapy and podiatry outpatient departments and (ii) whether these perceptions were influenced by health literacy. Methods: An opportunistic sample of people accessing services and healthcare professionals was obtained from physiotherapy and podiatry outpatient departments. People using services (n = 13) underwent in-depth interviews over Microsoft Teams. Healthcare professionals (n = 10) took part in two online focus groups. Thematic analysis using a framework approach was undertaken with codes applied to the “interdependent determinants of implementation success” framework. Results: Perceptions revolved around the characteristics of the implementation object (e.g., well laid out, short, concise, contained person-centred language), patient-level influences (e.g., concerns about delayed/withdrawal of care, reduced engagement as people faced other barriers to accessing and receiving healthcare) and collective-level influences (e.g., electronic data collection, automatic calculation of overall score, time-limited consultations, non-specific content, lack of training and culture of use). A comparison of the differences in perception with respect to health literacy (marginal and adequate only) was limited due to the small sample size, and therefore any findings are exploratory in nature. Conclusions: Preferred tools that could be used to support person-centred care were those that were short, concise, and well laid out and used person-centred wording but also contained enough detail to transfer important information from the people using services to healthcare professionals.

## 1. Introduction

There is an increasing movement towards person-centred models of care, whereby individuals are equal partners with their healthcare professionals, in deciding what matters the most and what investigations and interventions are appropriate [1]. There are significant benefits to taking a person-centred approach, including improving health outcomes, the retention of patient dignity, healthcare efficiencies and healthcare quality and safety [2,3]. One particular aspect of person-centred care is the appropriate use of language, with its core focus being to “maintain integrity of the individuals as human beings and to avoid language that objectifies a person by his or her condition” [4].

Patient-completed tools such as Patient-Reported Outcome Measures (PROMs), Patient-Reported Experience Measures (PREMs) and stratification (or empowerment) tools support the movement towards person-centred care. PROMs assess patient outcomes, and PREMs focus on the patient experience, while stratification tools address activation or self-management capacities. Other tools, which are often presented in the form of a short questionnaire, often focus on understanding the level of individual agency and one’s capabilities and enable healthcare staff to support self-management and provide the right level of care to meet a person’s needs. There is a need for a buy-in for these tools from people using services, healthcare professionals and healthcare managers for their use to be effective and provide the necessary information to have a positive impact. Buy-in could be viewed through the acceptability, engagement and uptake of interventions, which are influenced by the contextual factors that are necessary conditions and driving forces for implementation [5]. However, they can present a burden to patients and healthcare professionals, due to a lack of time and perceived irrelevance, leading to low engagement [6].

Despite patient empowerment and health literacy being intrinsically linked and forming part of person-centred healthcare models, they are two constructs that should be considered distinct within healthcare interventions [7]. Most of the literature on the tools designed to measure empowerment and self-efficacy focus on the validity of the tools, with some evidence to suggest that they are valid with different health literacy levels [8], rather than the uptake and engagement of tools implemented into routine care. Furthermore, interventions often focus on specific populations, health conditions or settings. This paper focuses on two allied healthcare professional specialities (podiatry and physiotherapy) with a broad population and types of health conditions, focusing on tools that are specifically designed to measure the empowerment and self-efficacy of individuals. Wales is rapidly implementing empowerment and self-efficacy tools, in conjunction with PROMs and PREMs, into routine care as part of the national Value-Based Healthcare agenda [9,10,11], the Healthier Wales strategy [12] and the National Clinical Framework [13]. However, for implementation to be effective, the perception of these tools among healthcare professionals and people using services needs to be considered to develop an implementation strategy that comes with the minimum burden but remains effective. Therefore, this study aims to document (i) the perception of tools that can measure empowerment and self-efficacy to inform person-centred care among healthcare professionals (physiotherapist and podiatrists) and people accessing physiotherapy and podiatry outpatient departments within a health board in Wales. A subsequent exploratory assessment was undertaken to see whether these perceptions were influenced by health literacy.

## 2. Materials and Methods

### 2.1. Theoretical Grounding

The “interdependent determinants of implementation success” is a determinant framework that includes the five types of determinants (characteristics of the implementation object, influences at the individual healthcare level, patient influence, collective-level influences, effectiveness of implementation strategies used to support the implementation) that are the main influences on implementation outcomes [14]. There are interdependencies between these five factors, but for this study they were used to identify and summarise the factors in which the participant’s perceptions are linked to help inform future implementation development. The framework was mainly used during the analysis stage; however, it did inform data collection through ensuring the inclusion of both people using services and healthcare professionals, as well as trialling the tools in practice.

### 2.2. Study Setting

This study was set in the physiotherapy and podiatry outpatient departments of one health board, within National Health Service (NHS) Wales. NHS Wales is a publicly funded healthcare system that provides healthcare that is free at the point of use. The physiotherapy and podiatry departments provide care for residents of the health board catchment area aged 18 years old and over. Within Wales, there is a movement towards the digitisation of healthcare, including digital patient-facing questionnaires and patient records. At the time this work was conducted, all members of the team were employed by the recruiting health board, R.L. as a physiotherapist and K.E.W. and K.W. as independently funded researchers. All were involved in the rapid review used to identify the tools [15].

### 2.3. Identification of Tools

A rapid review [15] and a professional advisory group, made up of healthcare professionals, NHS managers, researchers and PPI representatives, identified a range of tools (Table 1) that can be used by healthcare professionals to support people with self-management by measuring empowerment and self-efficacy. The professional advisory group identified the top three preferred tools from a pool of six top-scoring tools from the rapid review to use in this study based on the Psychometric and Pragmatic Evidence Rating Scale (PAPERS) [16] score and the fact that they were seen as patient-friendly and the most relevant to physiotherapy and podiatry. As the focus of this study is on empowerment and self-efficacy, PROMs and PREMs were not included within this study.

### 2.4. Recruitment

#### 2.4.1. People Using Services

**Figure 1:**
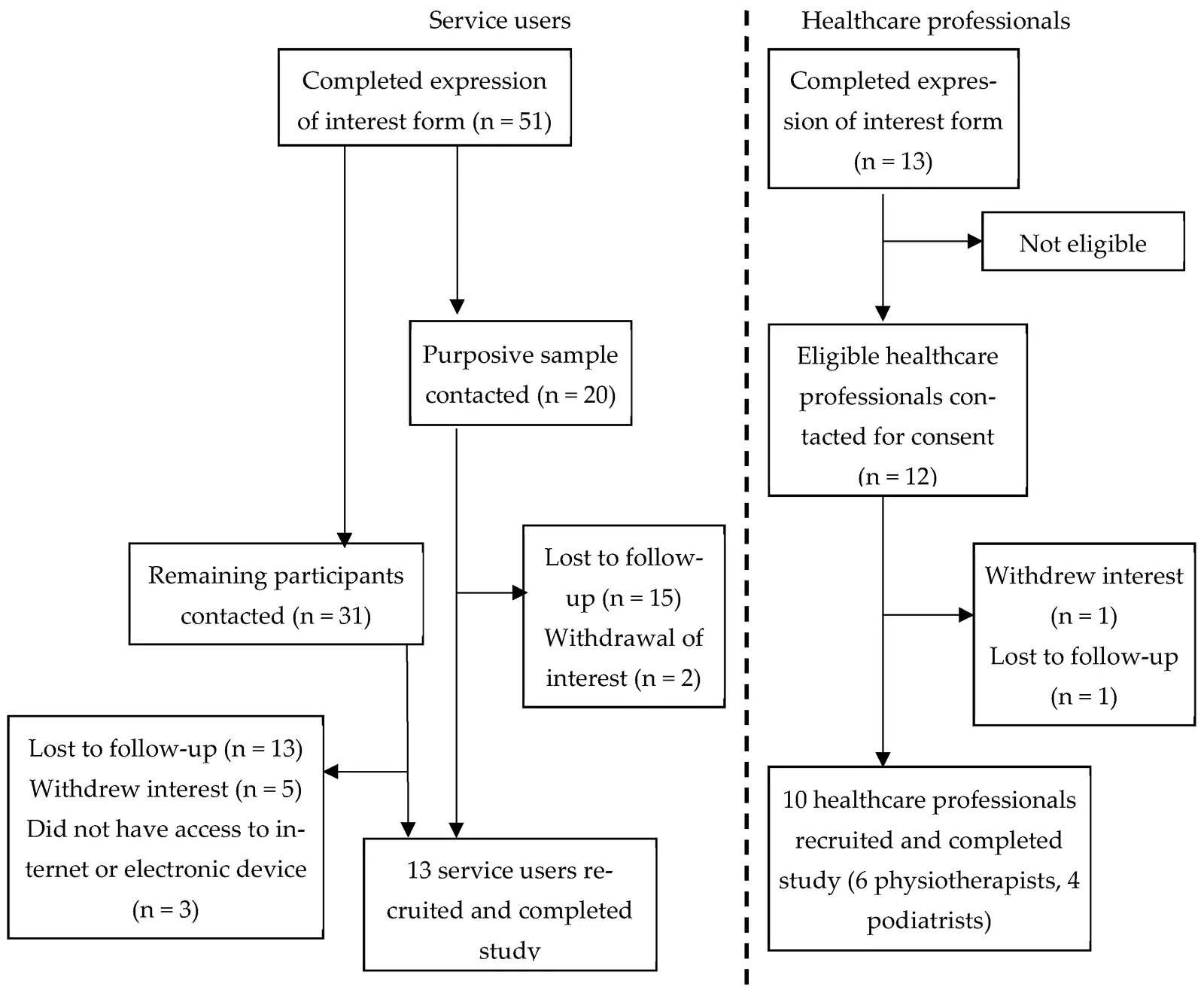
Flow diagram of participant recruitment.

People were recruited from the physiotherapy and podiatry outpatient clinics within one health board in NHS Wales, who were aged 18 years old and over, lived in Wales, had capacity and were attending an outpatient appointment. Interested participants were asked to fill out an expression of interest form, obtaining information on basic demographics, reason for appointment attendance, and health literacy level (determined using the BRIEF health literacy screen [20] (Supplementary Materials S1)). Implied consent was used, and participants were provided with a participant information sheet. A purposive sample was initially created based on health literacy and participant demographics. These participants were then invited to take part in an interview. However, due to some participants being lost to follow-up (n = 28) or withdrawing their expression of interest (n = 7), the final sample was opportunistic, comprising all remaining participants who responded to the interview invitation (Figure 1). Reasons for withdrawal included not having access to the internet or an electronic device or no longer being interested in this study. The opportunistic sample reduced the number of potential participants with lower health literacy scores, thus reducing comparability. The participant information sheet informed participants that their usual care would not be affected.

#### 2.4.2. Healthcare Professionals

Registered healthcare professionals, namely physiotherapists and podiatrists, working within the physiotherapy and podiatry departments with knowledge/experience of using PAM-13 were eligible to take part. The rationale for this eligibility criterion was due to the permissions around the use of PAM-13. Additionally, not all members of the physiotherapy department had lived experience of PAM-13, as it was not routinely used, and therefore including them could have risked unbalancing the sample. Additionally, PAM-13 was used in the two years prior to this study within the podiatry department, but the licence had expired by the start of this study. First contact was made using departmental email lists, sending an introductory email which contained a participant information sheet and an expression of interest form. An opportunistic sample was created based on those who returned an expression of interest form and met the inclusion criteria during screening.

### 2.5. Data Collection

Thirteen in-depth interviews were conducted with people using services, by K.E.W. alone over Microsoft Teams, and were structured using a topic guide (Supplementary Materials S2). All participants met K.E.W (a female researcher) in person when expressing interest to take part, and a rapport was developed during the contact made prior to the interview (i.e., interview invitation, answering questions, taking consent). All interviews (one per participant) took place on a weekday between 09:30 and 18:00 and lasted between 12 and 48 min. Participants were asked to digitally view or complete three tools prior to the interview. All three tools were also displayed at relevant points during the interviews, using the “share screen” function and in a standardised way (i.e., in the order according to the topic guide and in the same format). The tool outcomes were not used as part of this study, nor were the participants asked to report when they completed the tools in relation to the interview. All interviews were audio-recorded only (i.e., no field notes) and transcribed verbatim.

Two focus groups run by K.E.W. alone were undertaken with healthcare professionals; one group contained four participants (three physiotherapists and one podiatrist), and the other had six participants (three physiotherapists and three podiatrists). The focus groups took place during the working day on Microsoft Teams, lasted 80 min and were structured using a topic guide (Supplementary Materials S3). Staff trialled two tools (CHAI-7 and PEI-2) on 4–8 patients during their outpatient clinics (one tool per patient). For example, out of 8 patients, 4 used CHAI-7, and 4 used PEI-2. Overall, a total of 51 patients trialled the tools, with an almost equal split between CHAI-7 (n = 25) and PEI-2 (n = 26). Staff identified patients over the age of 18 years from their clinic lists and contacted them in advance of their appointments to provide the participant information sheet if they agreed to take part. Informed consent from these patients was taken by K.E.W., and tools were completed on a tablet. Staff were free to use the tool results within their consultation if clinically appropriate, but the tool outcomes were not used, entered in the research database or reported as part of this study. The reason for trialling the tools was so that the staff could use the tools to provide practical feedback during the focus group, not the patients who completed them. Staff did not trial PAM-13, as they already had prior experience of using it in clinical practice (see Section 2.4.2 for details). The healthcare professionals had not met K.E.W. before this study; therefore, rapport was built in the activities prior to the focus group. The focus groups were audio-recorded only (i.e., no field notes) and professionally transcribed verbatim by an external transcription company.

Confidentiality was maintained through the anonymisation of transcripts at the earliest opportunity, and the transcripts was stored on a secure server accessible only to the study team. The numbering system for participants is based on the allocation at the expression of interest, and therefore the maximum number is greater than the number of participants participating in the interviews.

### 2.6. Analysis

Thematic analysis with a framework approach [21,22] was undertaken by K.E.W. using Microsoft Word. Initial codes were generated while reading through and becoming familiar with (i.e., repeat reading of transcripts) the transcripts on MS Word. These codes from both the interviews and focus groups were integrated together and subsequently grouped and allocated into the relevant determinants of the pre-defined framework the “interdependent determinants of implementation success” based on their definitions [14]. The main reason for this was to understand and link perceptions to implementation development, but this could have featured within assumptions during analysis. However, reflexivity was maintained using a diary and a codebook. K.E.W., the lead researcher in this study, has extensive mixed methods experience in researching and evaluating complex interventions in public health and healthcare settings, with a background in applied health research. Two randomly selected interview transcripts were checked by another senior researcher independent of this project to identify discrepancies in the coding and identification of potential bias [23]. Only a few discrepancies were identified (i.e., allocation of a code from the codebook to a section of the transcript), which were resolved through verbal discussion. A collaborative determinant review was undertaken by K.E.W. and K.W. to maintain distance from those involved in implementation. The review did not identify any issue with the allocated codes to determinants and concluded that there was satisfactory alignment with the framework. Subsequently, all transcripts were re-read and re-coded where required. Transcripts and/or analysis findings were not returned to participants. Data saturation was assessed through tracking new codes across both the interview and focus group for the whole of their respective sample. Data saturation did not apply to the health literacy comparisons. Gender was defined as identification or presentation as women, men, or non-binary identities. The ranking of the tools from 1 to 10 within the interviews was not reported as it was used as an interview technique only, as the focus of this study was around the factors that make up preferences. All reporting followed the COREQ checklist [24].

### 2.7. Ethical Approval

Ethical approval was obtained from Northwest—Haydock Research Ethics Committee, via a proportionate review (IRAS: 330875). Cardiff and Vale University Health Board acted as a sponsor for this study (Sponsor ref: 8739). Informed consent for the interview, focus groups and trialling of the tools was obtained from all participants (healthcare professionals and people using services), and they were free to withdraw at any point. Implied consent from the expression of interest was inferred on the premise that the completion of the survey indicated that they consented to their responses and personal information being stored and handled, in line with current data protection legislation.

### 2.8. Participant and Public Involvement

A professional advisory group, including two participant and public involvement (PPI) representatives, was used as part of the project development phase. The PPI representatives also reviewed the study protocol and all participant-facing documents prior to submission for ethical approval.

## 3. Results

A total of 13 interviews were conducted, with seven people attending physiotherapy and six attending the podiatry department. The sample was predominantly female (n = 9; 69%) and had an “adequate” health literacy score (n = 9; 69%). None of the participants had a “limited” health literacy score. The average age was 58 years old (standard deviation: 14.4), with a range of 28–77 years old, and one participant’s first language was not English.

Participating healthcare professionals included six physiotherapists and four podiatrists across the two focus groups. All participants were experienced healthcare professionals, with a few participants having a level of seniority. Individual-level participant information was not reported due to the information being disclosive.

### 3.1. Characteristics of Implementation Object

Both people using services and healthcare professionals commented on the length, word type, and layout, with a preference towards shorter and concise elements that were easy to read and understand, as this was preferable for accessibility and engagement factors (i.e., PEI-2 and CHAI-7). Poor wording was seen as having a negative impact in terms of misinterpretation, less helpful conversations and creating a power imbalance between the person using the service and the healthcare professional (i.e., PEI-2 and PAM-13). In addition, it was suggested that engagement could be improved by adding context at the start of the tools, as there were concerns that the tools were too generic, which caused difficulty in identifying how completing the tool was linked to the current care being received. This was particularly noteworthy for people who have multiple chronic conditions or healthcare contacts. However, both people using services and healthcare professionals valued the provision of information and felt that it could build relationships. Healthcare professionals did strongly prefer terminology and content that focused on the person-centred model of healthcare, rather than the medical model. In terms of layout, the majority of people using services (n = 8) preferred tools that had answer options as “tick boxes” (i.e., PEI-2 and CHAI-7), as this was reported to be easier to understand and respond to and had greater visual appeal. Healthcare staff were hesitant to rank the tools within the focus groups as they felt that the tools did different jobs from screening (PEI-2) to intervention (PAM-13).

*“Sometimes it’s difficult getting the patients to engage, especially if there’s a long list of questions on the form”.*

*(Healthcare professional 12: Female, Podiatrist)*

*“It’s [PEI-2] straightforward, it’s simple, and it’s one that you would remember”.*

*(People using services 10: Female, Adequate health literacy)*

*“I think if you’ve got multiple issues sometimes perhaps it needs to be more clear as to well, which particular illness?”*

*(People using services 4: Female, Adequate health literacy)*

*“A bit more context before they answer I think, so it just gives them, I just find that sometimes they come in and we’ve all got busy lives, and they’re kind of on, they’re kind of linear thinking and, you know, it’s just nice, especially having time as well”.*

*(Healthcare professional 10: Female, Podiatrist)*

*“[I] Think it [tick boxes] is a lot more user friendly and less like kind of well that’s a lot of like information like straight away”.*

*(People using services 23: Male, Adequate health literacy)*

*“The questions were short [PEI-2]. I thought the language was clear and I like the answer options.”*

*(People using services 4: Female, Adequate health literacy)*

*“PEI-2 and CHAI-7 are screening tools for me, and the PAM-13 is an interventional tool.”*

*(Healthcare professional 3: Female, Physiotherapist)*

### 3.2. Patient Influence

Positive and negative underlying assumptions influenced people’s perception of the tools, as they made comments pertaining to how relevant the tools were to their care and the potential impact they may have on the receipt of care. For example, there were a few (n = 2) concerns that having a high activation level would potentially lead to care being delayed or withdrawn. Furthermore, some participants (n = 6) reported that they would give a lower score than they probably should have as they doubted themselves and perceived that they did not have “full” knowledge, despite coming across as knowledgeable and engaged people. Importantly the perception of the tools was influenced by the fact that some participants (n = 3) felt that there were greater barriers to accessing and receiving healthcare, which outweighed their understanding or ability to self-manage their health condition, thus reducing the importance of engaging with these tools.

*“I don’t think it’s [PEI-2] relevant to Podiatry, but if I was in for some something else, yes………. Podiatry is about the feet, not how I feel”*

*(People using services 8, Female, Marginal health literacy)*

*“But they’re all things that are out of my control in some sense. Because I want to be in control. And this is what this is all about. And therefore, that’s why I think some of these [PEI-2 questions] made me feel quite negative because I thought, oh, I’m doing absolutely everything in order to do it. I’ve sought the right help, etcetera, etcetera. But actually, there’s a point at which is it going to make a difference?”*

*(People using services 16: Female, Adequate health literacy)*

*“More comforted in the fact that, yeah, you’re getting more background information and more understanding of the patient’s needs.”*

*(People using services 20: Male, Adequate health literacy)*

### 3.3. Collective-Level Influences

Collective-level influences revolved around the facilitators and barriers to use, as reported by healthcare professionals. Facilitators to use in clinical practice included electronic data collection and the automatic calculation of the overall score (provided alongside the individual question answers). However, there were a few reports (n = 3) that there was a lot of information contained within the tools, making it hard to take in and digest it in a short amount of time in already time-limited consultations. Conversely, despite this limited time, there were reports (n = 2) that the tools enabled a quicker assessment of a patient’s empowerment level, which normally takes a while to determine within a consultation. Having non-specific content within the tools was seen as a barrier to use, as it opened conversations or issues that were not relevant to the specific care being provided in that consultation, which could make staff feel “out of their comfort zone”. Local pathways, training and generating a culture of use were reported as potential solutions to these barriers.

*“I sometimes think that the wording of things can set up a less helpful conversation, if that makes sense. If it’s not again pulling us towards that collaboration and that power balance between therapist and, and the patient from the offset”.*

*(Healthcare professional 4: Female, Podiatrist)*

*“I did find it quite difficult to process the information right at the start of the session and then integrate into my consultation.”*

*(Healthcare professional 4: Female, Podiatrist)*

*“Some of the patients that I’ve been dealing with will have been going through some really nasty cancer treatment, and we’ve been kind of keeping their nails at bay sort of thing. So able to cope with life at the moment, then open the doors to me not really being able to 100% respond, because obviously I’m not aware of, you know, all of the ins and outs of their cancer treatments and things like that.”*

*(Healthcare professional 8: Female, Physiotherapist)*

*“The tool is used as part, a key part of the, the consultation, you know, it’s a vital piece of information to inform the consultation, and everybody realises and understands that, then your response rate goes up and interaction goes up”.*

*(Healthcare professional 1: Male, Physiotherapist)*

*“If they do come out as kind of lower activated, I just feel like um, as clinicians um, we don’t have enough training within our department to know how to really assist them through that.”*

*(Healthcare professional 8: Female, Physiotherapist)*

*“I think it’s a measure of what society expects of healthcare, and it’s the picture that we paint of healthcare that’s really important here.”*

*(Healthcare professional 1: Male, Physiotherapist)*

### 3.4. Role of Health Literacy

Differences in peoples’ perceptions between a “marginal” and “adequate” health literacy level was challenging to quantify as there was no singular determinant that clearly fell into one health literacy level. However, there were some emerging patterns that require further exploration, such as that people using services with adequate health literacy levels tended not to report issues with longer or more complex tools (i.e., CHAI-7 and PAM-13) and were more likely to comment on the content and overanalyse questions. One participant did note the challenge of translating complex wording from PAM-13 back into their own first language.

Healthcare professionals noted that health literacy may impact the completion of the tools, but other factors (e.g., general literacy levels, language, the worried-well, societal expectations) may also play a role. Throughout the discussion, staff balanced the need for obtaining useful information that supports clinical care being provided and the need for the tools to be accessible and appropriate for the general population.

## 4. Discussion

This study documented the perceptions and preferences of people using services and healthcare professionals in terms of the empowerment and self-efficacy measurement tools (questionnaires) used to support person-centred care. Participants generally saw potential value in such tools, despite concerns around potential negative impacts on care, limited time within consultations and other external barriers to healthcare. Using the “interdependent determinants of implementation success” framework, perceptions revolved around the characteristics of the implementation (e.g., short, concise, contained person-centred language, well laid out), patient-level influences (e.g., concerns about delayed/withdrawal of care, reduced engagement as patients faced other barriers to accessing and receiving healthcare) and collective-level influences (e.g., electronic data collection, automatic calculation of overall score, time-limited consultations, non-specific content, lack of training and culture of use). While some patient activation tools were preferred, participants’ preferences were rarely based on a single feature or characteristic. Instead, preferences were influenced by a range of contextual considerations. Although the allocation of code was explored in terms of the “individual healthcare level” and “strategies for implementation” domains, no relevant data emerged for these domains, which may be in part due to the questions in the topic guides being focused on the tools themselves rather than the mechanism of implementation and the fact that the framework was only applied during the analysis. Therefore, future research should address these gaps in evidence.

Having a balance between short and concise questionnaires, which contains pertinent and relevant information is documented within the literature, minaly in relation to the burden of use and time constraints [25]. This is particularly relevant in the context of the National Health Service, where resources are constrained and there is high demand. Staff did report the increased time demand of the tools on one hand but valued the ability to obtain information quickly on the other. Further investigation is needed to understand how validated questionnaires could aid clinical decision-making and resource allocation (i.e., shifting resources from individuals with high agency to those who might require more support) and its cost-effectiveness. However, this approach requires further investigation and should take into consideration the concerns raised by people using treatment services of being withdrawn or services being delayed if they have a high activation level. Implementation strategies must ensure that the right tools are chosen to facilitate engagement and that appropriate mechanisms are in place. This study also focused on the digital completion of tools, given the context of a shift to digital healthcare within Wales, and therefore did not address the potential need for alternative solutions for the digitally excluded. Further work will be required to ensure care pathways (e.g., signposting), the mechanism of use in care (e.g., screening tool, allocation of care), and training and developing a culture of use will minimise the burden on people using services and healthcare professionals and help achieve the intended effect. For example, any context given prior to the tool, as suggested by participants, should be carefully worded to avoid reporting biases [26]. If implemented successfully, these tools could have an impact by supporting healthcare professionals to deliver person-centred care and prevent under- or overtreatment with personalised treatment plans [27,28].

The divergence of opinions between people using services and healthcare professionals (e.g., use as a stratification tool, questionnaire content) may be due to previous lived experience (i.e., difficulty accessing healthcare), the participants’ role in the healthcare interaction (e.g., delivering versus receiving care), and differing degrees of exposure to the tools within this study. These differences are important to consider in any implementation strategy. Furthermore, the questionnaires were only provided in English, which may have impacted the ability of participants with a different first language to engage with this study and may have influenced their perceptions of the tools. Therefore, further research needs to be undertaken to explore the impact of non-native English speakers’ perception of the tools, both in their own language and using the English version of tools. In addition, unintended consequences need to be investigated, which could include, but are not limited to, widening inequalities in healthcare which may occur when there is not equitable access to tools [29], especially if these are accessed digitally [30]; the perception of negative impacts on care; and disempowerment [31].

In the context of the limitations of this study (i.e., small sample, measurement bias and participation bias), conclusions around having a health literacy level of “marginal” compared to “adequate” and the influence that people’s perceptions of the tools has were limited and therefore require further investigation. Findings from a previous study suggest that there is no association between health literacy and patient activation [25]. However, another study [26] documented variation in the level of health literacy, from functional to interactive to critical health literacy, with the latter level being a determinant of health-promotion success. It is therefore possible that the BRIEF health literacy screen may not have been sensitive enough to identify how health literacy may impact the perceptions of tools that measure patient activation. It is likely that preferences for and opinions on the tools were more likely to be influenced by other factors such as gender and age [32,33], as well as the subjective nature of the tools [26], which requires further investigation. Even though this study looked at perceptions, healthcare professionals did feel that health literacy levels play an important role in patient empowerment.

Although an attempt was made to ensure the sample included a range of health literacy statuses and demographics through purposive sampling, initial recruitment was low, and several participants who filled out an expression of interest form were lost to follow-up, resulting in a small, more opportunistic sample. Despite this, there was emerging evidence that data saturation had been met due to no new information being provided (i.e., no new codes) in the later interviews or between the two focus groups. The sample is likely to be skewed towards activated individuals, as they are more likely to participate in research activities, in addition to the sample being biased towards those with digital access (e.g., younger individuals, wealthier individuals, those with higher health literacy) [34]. Furthermore, some of the participants declared that they were current or retired healthcare professionals, even though they were not specifically asked. There was evidence to suggest that this may have impacted their perceptions of the tools, but this was not formally assessed across all participants and therefore requires further investigation. Similarly, participation bias was seen among staff, with more senior or experienced physiotherapists or podiatrists dominating the sample. This interpretation is based on the self-declared job roles within the focus groups but was not a formally assessed participant characteristic. Having prior experience with PAM-13 compared to the limited experience with CHAI-7 and PEI-2 obtained while testing the tool may have impacted staff views on implementation and acceptability, but this was a necessary compromise due to the challenges of using PAM-13. Further sample bias may be present due to the podiatrists having a greater level of experience with PAM-13, with it being used in routine care within the two years prior to this study, and therefore, they may have different views of usefulness and feasibility within routine care. The staff also selected people using services to test the tools that were more likely to participate and were at different points in the patient journey. This may have reduced the utility of the tools that measure patient activation and led to a selection bias. Any future work would need to effectively recruit a diverse range of participants to enable a successful comparison across different services, populations and regions. As with any qualitative research, a differing theoretical grounding, analysis method or researcher reflexivity may have yielded different results or limited emergent insights. However, the framework chosen did summarise valuable findings specific to implementation within a complex system that are hopefully informative for both research and the decision-making community.

## 5. Conclusions

Preferable empowerment and self-efficacy measurement tools for supporting person-centred care were short, concise, and well laid out and used person-centred wording but also contained sufficient detail for transferring important information from people using services to healthcare professionals. Both people using services and healthcare professionals see potential value in their use. In the context of the limitations of this study (i.e., small sample, measurement bias and participation bias), no conclusions can be made regarding differences in perception between “marginal” and “adequate” health literacy levels. Further work is required to co-design implementation strategies that minimise the risk of unintended consequences and widening of inequalities.

## Figures and Tables

**Table 1:** The empowerment and self-efficacy measurement tools identified within the rapid review [15], which were considered for use in this study.

Empowerment and Self-Efficacy Measurement Tool	Number of Questions	Response Options	Produces an Overall Score	Score Interpretation	Response Format	Construct
CHAI-7 *	7	6-point Likert scale	Yes (Transformed score 0–100)	Low activation: 0–79 Moderate activation: 80–94 High activation: 95–100 [8,17]	Tick boxes	Health activation
LTCQ	20	5-point Likert scale	No	-	Tick boxes	Living well with long-term conditions
LTCQ-8	8	5-point Likert scale	No	-	Tick boxes	Living well with long-term conditions
PAM-13 *	13	4-point Likert scale	Yes (Transformed score 0–100)	Level 1: Disengaged and overwhelmed (≤) Level 2: Becoming aware but still struggling (47.1–55.1) Level 3: Taking action (55.2–67) Level 4: Maintaining behaviours and pushing further (≥67.1) [18,19]	Circling words	Knowledge, skills and confidence in managing their health
PEI-2 *	6	5-point Likert scale	No	-	Tick boxes	Health enablement
SEMCD-6	6	0–10 scale from not at all confident to confident	No	-	Selection on a scale	Self-efficacy for managing chronic conditions

Abbreviations: CHAI = Consumer Health Activation Index; LTCQ = Long-Term Conditions Questionnaire; PAM = Patient Activation Measure; PEI = Patient Enablement Instrument (version 2); SEMCD = Self-Efficacy for Managing Chronic Disease; * included in this study. The full findings of the rapid review and PAPERS score are reported elsewhere [15].

## Data Availability

The data within this study is not publicly available due to confidentiality or ethical reasons.

## Supplementary Materials

The following supporting information can be downloaded at https://www.mdpi.com/article/10.3390/healthcare14213778/s1, Supplementary Materials S1: BRIEF health literacy screen. Supplementary Materials S2: Interview topic guide. Supplementary Materials S3: Focus group topic guide.

## Abbreviations

The following abbreviations are used in this manuscript:

NHS	National Health Service
CHAI	Consumer Health Activation Index
LTCQ	Long-Term Conditions Questionnaire
PAM	Patient Activation Measure
PAPERS	Psychometric and Pragmatic Evidence Rating Scale
PEI	Patient Enablement Instrument
PPI	Patient and Public Involvement
PREM	Patient-Reported Experience Measure
PROM	Patient-Reported Outcome Measure
SEMCD	Self-Efficacy for Managing Chronic Disease
