# Supplementary material for: “What Society Expects of Healthcare and the Picture That We Paint”—A Qualitative Piece in the Puzzle to Inform Implementation: What Do People Using Services and Healthcare Professionals Look for in Questionnaires to Support Person-Centred Care in Physiotherapy and Podiatry Outpatients?
Source: Healthcare (Basel). 2026 Nov 5;14(21):3778. doi: 10.3390/healthcare14213778 (PMC13763144; doi:10.3390/healthcare14213778)
Supplement: Supplementary file 1 [file healthcare-14-03778-s001.zip › Supplementary Materials S1.pdf]

Supplementary Materials S1: BRIEF health literacy screen

Sand-Jecklin K, Coyle S. Brief Health Literacy Screen (BHLS). APA PsycTests; 2014; Available from: https://doi.org/10.1037/t37729-000.

BRIEF: Health Literacy Screening Tool

Please circle the answer that best represents your response.

1. How often do you have someone help you read hospital materials?

1. Always
2. Often
3. Sometimes
4. Occasionally
5. Never

2. How often do you have problems learning about your medical condition because of difficulty understanding written information?

1. Always
2. Often
3. Sometimes
4. Occasionally
5. Never

3. How often do you have a problem understanding what is told to you about your medical condition?

1. Always
2. Often
3. Sometimes
4. Occasionally
5. Never

4. How confident are you filling out medical forms by yourself?

1. Not at all
2. A little bit
3. Somewhat
4. Quite a bit
5. Extremely

Clinicians can ask patients these four questions to determine their patients' health literacy level (the degree to which one can read, understand, exchange, and use health information and resources). Each item is worth 1 to 5 points depending on their response (as seen in numbers to the left of the answer options – i.e., response 1 = 1 point). Add the values for the four responses to get a total score, which can range from a minimum of 4 to a maximum to 20. To interpret scores refer to the following table:

**BRIEF**	**Score**	**Skills and Abilities**
Limited	4-12	Not able to read most low literacy health materials; will need repeated oral instructions; materials should be composed of illustrations or video tapes. Will need low literacy materials; may not be able to read a prescription label.
Marginal	13-16	May need assistance; may struggle with patient education materials.
Adequate	17-20	Will be able to read and comprehend most patient education materials
