# Supplementary material for: “What Society Expects of Healthcare and the Picture That We Paint”—A Qualitative Piece in the Puzzle to Inform Implementation: What Do People Using Services and Healthcare Professionals Look for in Questionnaires to Support Person-Centred Care in Physiotherapy and Podiatry Outpatients?
Source: Healthcare (Basel). 2026 Nov 5;14(21):3778. doi: 10.3390/healthcare14213778 (PMC13763144; doi:10.3390/healthcare14213778)
Supplement: Supplementary file 1 [file healthcare-14-03778-s001.zip › Supplementary Materials S2.pdf]

Supplementary Materials S2: Interview topic guide

a) I am going to run through three tools with you now. At then after each tool I would like you to rank them out of 10, where 1 is the worst possible score and 10 is the best possible score:
   a. How easy were the questions to understand?
   b. The number of questions (1 = too short, 10 = too long)
   c. Thinking about your current care were the questions relevant to the care you receive?

b) Now that you have been through each of the tools, would you change the score you originally gave them? [remind participant of scores]

Now we have completed all the tools, can you run through the positives and negatives for each tool.

c) What were your thoughts and opinions on tool 1?
   a. Positive
   b. Negative

d) What were your thoughts and opinions on tool 2?
   a. Positive
   b. Negative

e) What were your thoughts and opinions on tool 3?
   a. Positive
   b. Negative

f) Can you rank the tools in order of preference? Why have you chosen that ranking?

g) Do you think these tools would be useful in the care you receive? Why?

Thank you for spending the time to talk to me today. Is there anything else you would like to tell me?
