# Supplementary material for: “What Society Expects of Healthcare and the Picture That We Paint”—A Qualitative Piece in the Puzzle to Inform Implementation: What Do People Using Services and Healthcare Professionals Look for in Questionnaires to Support Person-Centred Care in Physiotherapy and Podiatry Outpatients?
Source: Healthcare (Basel). 2026 Nov 5;14(21):3778. doi: 10.3390/healthcare14213778 (PMC13763144; doi:10.3390/healthcare14213778)
Supplement: Supplementary file 1 [file healthcare-14-03778-s001.zip › Supplementary Materials S3.pdf]

Supplementary Materials S3: Focus group topic guide

a) Can each of you give me a brief introduction to your current role and experience?
b) Have you ever used patient empowerment tools before this study? If yes, in what context and how confident are you in using them?
c) What were your thoughts and opinions on tool 1?
    a. Positive
    b. Negative
d) What were your thoughts and opinions on tool 2?
    a. Positive
    b. Negative
e) What were your thoughts and opinions on tool 3?
    a. Positive
    b. Negative
f) How would you rank the tools and why?
g) When we talk about health literacy in this study, we mean "the personal characteristics and social resources needed for individuals and communities to access, understand, appraise and use information and services to make decisions about health. Health literacy includes the capacity to communicate, assert and enact these decisions"[1] . How do you think the health literacy level of a patient would impact each of the tools?
h) Would any of these tools be useful for your practice? Why?

Thank you for spending the time to talk to me today. Is there anything else you would like to tell me?

[1] https://library.nhs.uk/wp-content/uploads/sites/4/2020/08/Health-literacy-how-to-guide.pdf
